# Glucose substitution prolongs maintenance of energy homeostasis and lifespan of telomere dysfunctional mice

**DOI:** 10.1038/ncomms5924

**Published:** 2014-09-18

**Authors:** Pavlos Missios, Yuan Zhou, Luis Miguel Guachalla, Guido von Figura, Andre Wegner, Sundaram Reddy Chakkarappan, Tina Binz, Anne Gompf, Götz Hartleben, Martin D. Burkhalter, Veronika Wulff, Cagatay Günes, Rui Wang Sattler, Zhangfa Song, Thomas Illig, Susanne Klaus, Bernhard O. Böhm, Tina Wenz, Karsten Hiller, K. Lenhard Rudolph

**Affiliations:** 1 Cooperation Group of the Leibniz Institute for Age Research—Fritz-Lipmann-Institute (FLI) Jena with the University of Ulm, 89081 Ulm, Germany; 2 Luxembourg Centre for Systems Biomedicine, University of Luxembourg, 7, avenue des Hauts-Fourneaux, Esch-Belval L-4362, Luxembourg; 3 Leibniz Institute for Age Research—Fritz Lipmann Institute (FLI), Beutenbergstr 11, 07745 Jena, Germany; 4 Institute for Genetics, Excellence Cluster on Cellular Stress Responses in Aging-Associated Diseases (CECAD), University of Cologne, Zülpicher Street 47A, 50674 Cologne, Germany; 5 Institute of Epidemiology, Ingolstädter Landstrasse 1, 85764 Munich/Neuherberg, Germany; 6 German Institute of Human Nutrition, Arthur-Scheunert-Allee 114-116, 14558 Nuthetal, Germany; 7 Department of Internal Medicine I, University of Ulm, 89081 Ulm, Germany

## Abstract

DNA damage and telomere dysfunction shorten organismal lifespan. Here we show that oral glucose administration at advanced age increases health and lifespan of telomere dysfunctional mice. The study reveals that energy consumption increases in telomere dysfunctional cells resulting in enhanced glucose metabolism both in glycolysis and in the tricarboxylic acid cycle at organismal level. In ageing telomere dysfunctional mice, normal diet provides insufficient amounts of glucose thus leading to impaired energy homeostasis, catabolism, suppression of IGF-1/mTOR signalling, suppression of mitochondrial biogenesis and tissue atrophy. A glucose-enriched diet reverts these defects by activating glycolysis, mitochondrial biogenesis and oxidative glucose metabolism. The beneficial effects of glucose substitution on mitochondrial function and glucose metabolism are blocked by mTOR inhibition but mimicked by IGF-1 application. Together, these results provide the first experimental evidence that telomere dysfunction enhances the requirement of glucose substitution for the maintenance of energy homeostasis and IGF-1/mTOR-dependent mitochondrial biogenesis in ageing tissues.

## Abstract (web summary)

Shortened telomeres and reduced mitochondrial biogenesis are cellular hallmarks of ageing. Here, Missios *et al.* show that old mice with telomere dysfunction have an increased energetic demand that cannot be met unless mice are fed a glucose-rich diet, which improves energy metabolism and extends lifespan.

## Graphical abstract

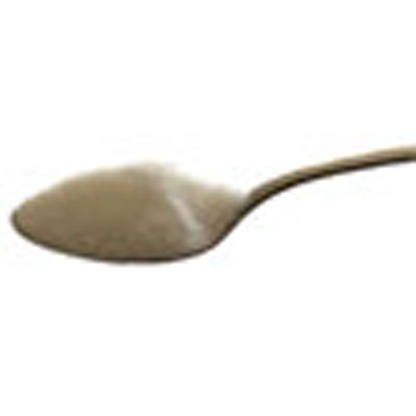

The accumulation of DNA damage and telomere dysfunction occurs during mouse and human ageing. The activation of DNA damage checkpoints impairs organ maintenance and lifespan in response to telomere dysfunction by inhibiting stem cell self-renewal as well as proliferation and survival of regenerative cells^1^,^2^,^3^,^4^,^5^. There is emerging evidence that telomere dysfunction and DNA damage impair mitochondrial biogenesis through p53/p21 activation, and that this mechanism may contribute to tissue ageing in response to DNA damage accumulation^6^,^7^. Recent publications revealed that cellular senescence in response to oncogene activation increases the energy expenditure of cells^8^,^9^. Senescent cells exhibit increases in glucose metabolism in the tricarboxylic acid (TCA) cycle and elevated levels of ATP. Whether similar responses occur in ageing cells and tissues in response to DNA damage or telomere dysfunction-induced senescence is currently unknown.

In DNA repair-deficient mice, the accumulation of DNA damage is associated with the suppression of insulin/insulin-like growth factor 1 (IGF-1) signalling; it has been suggested that the downregulation of the somatotrophic axis represents an adaptive response to allocate energy to cellular repair instead of proliferation in the context of DNA damage^10^. In agreement with these interpretations, reduction in the mammalian target of rapamycin (mTOR) and insulin/IGF-1 signalling mediate lifespan extension by dietary restriction in *Caenorhabditis elegans* and *Drosophila*^11^,^12^,^13^,^14^,^15^,^16^, and both pathways influence mammalian ageing^17^,^18^. However, inhibition of mTOR and IGF-1 signalling can also result in decreased mitochondrial function in mammalian cells^15^,^19^. Based on the findings that telomere dysfunction impairs mitochondrial functionality and that induction of senescence increases the energy demand of cells, it is possible that the accumulation of DNA damage would also lead to changes in dietary requirements of ageing tissues.

Here we analysed consequences of glucose substitution on metabolism and lifespan of telomerase knockout (KO) mice with dysfunctional telomeres. The study shows that telomere dysfunction increases the energy demand of telomere dysfunctional cells and glucose content of normal diet becomes limiting for the maintenance of energy homeostasis at the organismal level. In this context, the substitution of glucose leads to a significant extension of the lifespan of the mice by stimulating glycolysis, IGF-1/mTOR-dependent mitochondrial biogenesis and oxidative glucose metabolism.

## Results

### Glucose-enriched diet rescues lifespan of G3 *mTerc* KO mice

**Figure 1:**
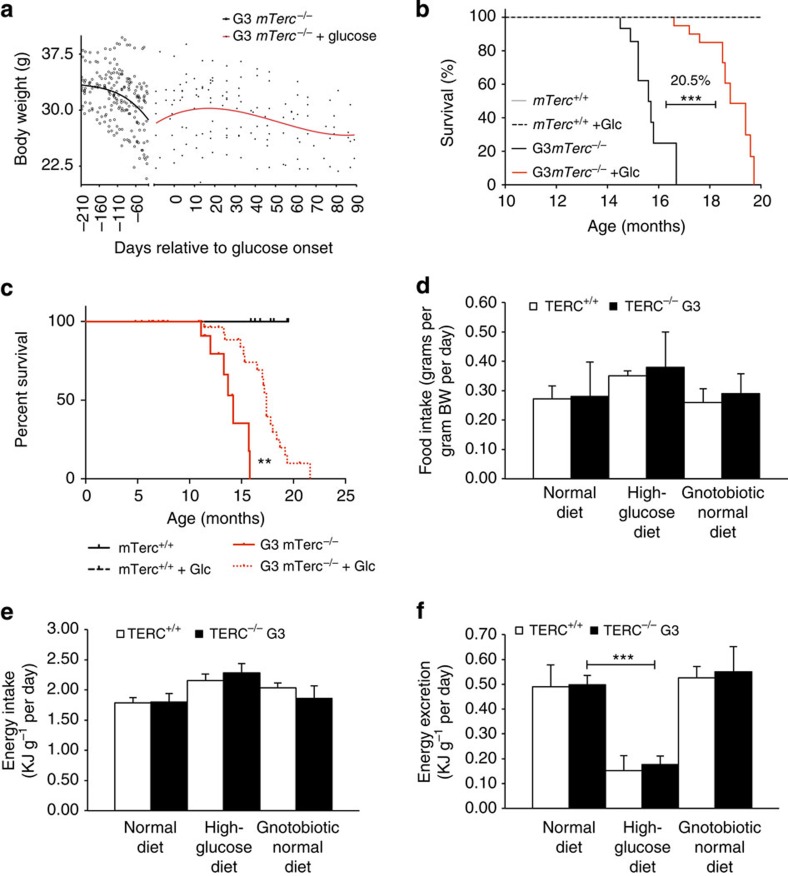
Glucose feeding extends lifespan of telomere dysfunctional mice. (**a**) G3 *mTerc*^−/−^ mice (12- to 15-month old) were fed with a glucose-rich diet when mice lost 10–15% of body weight on normal *ad libitum* diet (*n*=13 mice). The dot plot shows body weights of G3 *mTerc*^*−*/−^ mice at the indicated time points before (black line) and after switching the mice from a normal to a glucose-enriched diet (red line). (**b**) Survival curves of *mTerc*^*+/+*^ and G3 *mTerc*^*−*/−^ mice under the different diets. G3 *mTerc*^−/−^ mice (12- to 15-month old) that exhibited weight loss on normal diet were shifted to a glucose-enriched diet (red line) or continuously fed with normal diet (black line). (**c**) Kaplan–Meier survival curve: *mTerc*^*+/+*^ and G3 *mTerc*^*−*/−^ mice under the different diets. G3 *mTerc*^−/−^ mice (13.5-month old), which did not yet exhibit weight loss on normal diet, were shifted to a glucose-enriched diet (red dotted line) or continuously fed with normal diet (red straight line) (*n*=50 mice). Log-rank test, **=*P*<0.01. (**d**–**f**) Analysis of daily food intake normalized to body weight (*n*=9–11 mice per group). (**d**) Food intake in relation to body weight in grams per day. Energy intake (**e**) as well as energy excretion (**f**) in relation to the body weight of the respective groups per day. All statistical data were assessed using Student’s *t*-test and are presented as mean±s.d. WT, wild type. *P<0.05, **P<0.01, ***P<0.001.

Owing to long telomere reserves in the C57Bl/6J inbred mouse strain, homozygous deletion of the RNA component of telomerase (*mTerc*) does not induce strong phenotypes in first-generation KO mice (G1 *mTerc*^−/−^). When homozygous *mTerc* KO mice are crossed to each other through successive generations, the shortening of telomeres from one generation to the next leads to telomere dysfunction and premature ageing in third-generation KO mice (G3 *mTerc*^*−*/−^). G3 *mTerc*^*−*/−^ mice have a normal body weight at birth and young adulthood, but exhibit a premature decrease in body weight during ageing when compared with *mTerc*^+/+^ mice^2^. At the time the body weight loss of aged G3 *mTerc*^*−*/−^ mice reaches 10–15%, survival of the mice is limited to 2–4 weeks, and the mice have to be killed because of a rapid decrease in fitness and appearance of a moribund phenotype (Fig. 1a,b). This premature weight loss is associated with premature ageing of organ systems with high rates of cell turnover^2^ and a shortened survival compared with *mTerc*^*+/+*^ mice with long telomere reserves (Fig. 1b,c).

Analysis of weight-adjusted food intake rates of 12- to 15-month-old G3 *mTerc*^*−*/−^ mice (with weight loss on normal diet) and age-matched *mTerc*^+/+^ controls revealed no significant differences in food/energy intake between the cohorts (Fig. 1d,e). In addition, calorimetric determination of faecal energy content of the same mice showed similar rates of energy excretion of 12- to 15–month-old G3 *mTerc*^*−*/−^ mice compared with age-matched *mTerc*^+/+^ mice (Fig. 1f). Similar results were obtained for 12- to 15-month-old gnotobiotic (microbial free) G3 *mTerc*^*−*/−^ mice exhibiting signs of premature ageing (Fig. 1d–f). Together, these results showed that differences in energy uptake were not responsible for the premature weight loss and the shortened lifespan of G3 *mTerc*^−/−^ mice compared with *mTerc*^+/+^ mice on normal diet (Fig. 1a–c).

To test whether increases in energy uptake by feeding of a glucose-enriched diet would influence the survival of mice with dysfunctional telomeres, G3 *mTerc*^*−*/−^ mice were switched to a glucose-rich diet containing an elevated total content of carbohydrates that contain glucose instead of long-chain carbohydrates, but with the same total amount of energy compared with normal diet (Supplementary Table 1). Specifically, two experiments were conducted: 12- to 15-month-old G3 *mTerc*^−/−^ mice that exhibited 10–15% body weight loss on normal diet were shifted to a glucose-enriched diet or continuously fed with normal diet (Fig. 1a,b). In a second experiment, a cohort of 13.5-month-old G3 *mTerc*^−/−^ mice that did not yet exhibit weight loss on normal diet were shifted to a glucose-enriched diet or continuously fed with normal diet (Fig. 1c). In the first experiment, the exposure of 12- to 15-month-old G3 *mTerc*^*−*/−^ mice (that lost body weight on normal diet) to a glucose-enriched diet led to an increase in body weight and elongated the lifespan of the mice by 20.5% compared with age-matched G3 *mTerc*^−/−^ mice that were kept on normal diet (Fig. 1a,b). In the second experiment, the exposure of 13.5-month-old G3 *mTerc*^−/−^ mice (without body weight loss) to a glucose-enriched diet led to a significant elongation in lifespan compared with an age-matched control cohort of matched G3 *mTerc*^−/−^ mice that were kept on normal diet (Fig. 1c). Together, these results provided the first experimental evidence that glucose substitution extends the lifespan of aged G3 *mTerc*^−/−^ mice. In both cohorts (*mTerc*^+/+^ and G3 *mTerc*^−/−^), the glucose-enriched diet showed significantly higher digestibility rates compared with normal diet (Supplementary Fig. 1A), indicating that the glucose-enriched diet increased the energy uptake in both cohorts.

**Figure 2:**
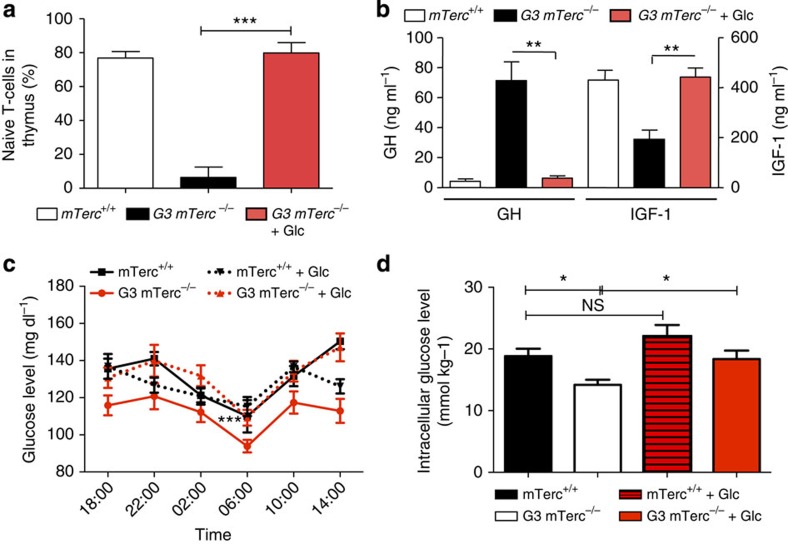
Glucose supplementation rescues thymus atrophy and IGF-1 and glucose levels of telomere dysfunctional mice. 12–15 month old G3 *mTerc*^−/−^ mice with weight loss on normal ad libitum diet and age-matched *mTerc*^+/+^ mice were analyzed under continuous exposure to normal ad libitum diet or 2 weeks after switching to a glucose enriched diet (+Glc): (**a**) Percentage of double-positive CD4^+^/CD8^+^ naive T cells in thymus (*n*=3 per group). (**b**) Blood plasma levels of IGF-1 and growth hormone (GH) in ng ml^−1^ (*n*=4–7 mice per group). (**c**) Diurnal plasma glucose levels were measured every 4 h (*n*=4–5 mice per group). G3 *mTerc*^−/−^ mice (12- to 15-month old) on normal diet exhibit significantly lower glucose levels compared with all other groups (*P*<0.001). Glucose levels were measured in mg dl^−1^. (**d**) Intracellular glucose levels in liver (*n*=4–5 mice per group). All statistical data were assessed using Student’s *t*-test and are presented as mean±s.e.m. NS, non–significant. *P<0.05, **P<0.01, ***P<0.001.

The premature involution of the thymus represents one of the prominent phenotypes of premature ageing in telomere dysfunctional mice^20^. Glucose supplementation rescued premature thymus involution and the maintenance of T-lymphocyte progenitor cells (CD4/CD8 double-positive) in the thymus of 12- to 15-month-old G3 *mTerc*^−/−^ mice with weight loss compared with age-matched *mTerc*^+/+^ mice (Fig. 2a). In addition, glucose substitution of moribund G3 *mTerc*^−/−^ mice resulted in quick and overt improvements in organismal fitness (data not shown).

**Figure 3:**
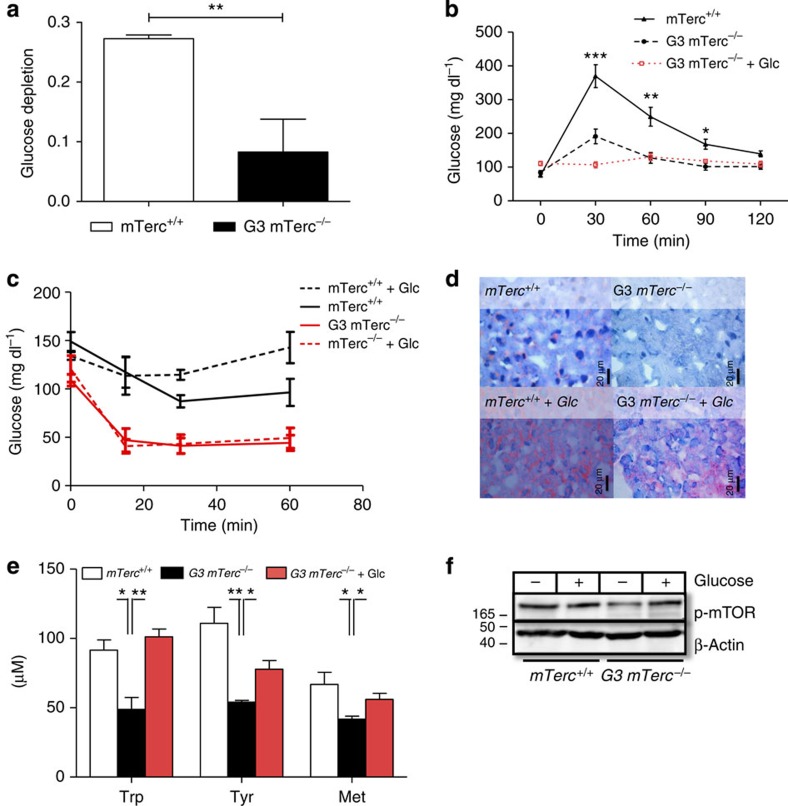
Telomere dysfunctional mice show elevated glucose depletion rates and glucose supplementation rescues catabolic metabolism in this context. 12–15 month old G3 *mTerc*^−/−^ mice that exhibited (**a**,**c**–**f**) weight loss on normal ad libitum diet or a regane in body weight (2 weeks after initiation of a glucose enriched diet) or (**b**) a reappearance of body weight loss 2 month after initiation of the glucose enriched diet were compared to age-matched *mTerc*^+/+^ mice. (**a**) Ratio of U^13^C-labelled glucose at 180 min versus 10 min after i.p. injection of labelled glucose (*n*=3 mice per group). (**b**) Glucose tolerance test. The histogram shows blood glucose levels in mg dl^−1^ after a bolus injection of glucose after overnight fasting (*n*=4–9 mice per group). (**c**) Insulin tolerance test. The histogram shows blood glucose levels in mg dl^−1^ after a bolus injection of insulin (*n*=4–9 mice per group). (**d**) Representative pictures of Oil-red-O fat staining in liver sections. Red droplets indicate hepatic fat storage. (**e**) Serum levels of the essential amino acids (tryptophan (Trp), tyrosine (Tyr) and methionine (Met)) in aged mice with and without glucose supplementation (*n*=3 pools per group, three mice per pool). (**f**) The western blot analysis shows the expressions of phosphorylated mTOR in liver tissues of mice of the indicated genotype, fed with *ad libitum* normal diet or high-glucose diet (*n*=4–5 mice per group). All statistical data were assessed using Student’s *t*-test and are presented as mean±s.e.m. *P<0.05, **P<0.01, ***P<0.001.

Young, telomere dysfunctional mice (2- to 4-month old) were shown to have elevated plasma glucose levels, which appears to be due to delayed postnatal growth of the pancreatic islets^21^. However, this phenotype disappears at mid-age (6- to 8-month old) when glucose levels are normal in G3 *mTerc*^*−*/−^ mice compared with wild-type mice. At the age of 12–15 months, telomere dysfunctional mice (G3 *mTerc*^*−*/−^) develop a progressive weight loss on normal diet and then exhibit several characteristic features of fasting. The mice show a suppression of IGF-1 levels accompanied by an elevation of growth hormone (GH) levels (Fig. 2b). Such an uncoupling of elevated GH levels without induction of IGF-1 is reminiscent of previously characterized fasting response mediated by induction of fibroblast growth factor 21 (FGF-21)^22^. In addition, ageing telomere dysfunctional mice showed increased expression of the starvation response protein insulin-like growth factor-binding protein 1 (IGFBP-1) in liver (Supplementary Fig. 1B), reduced glucose levels in blood (Fig. 2c) and liver (Fig. 2d), much faster kinetics in the depletion of labelled glucose in blood and liver after bolus injection of overnight-starved mice (Fig. 3a and Supplementary Fig. 1C), pathologically enhanced clearance of glucose in the glucose tolerance test and increased insulin sensitivity (Fig. 3b,c), decreases in hepatic fat content (Fig. 3d) and glycogen storage (Supplementary Fig. 1D), reduced levels of essential amino acids in blood serum (Fig. 3e) and diminished expression of phosphorylated mTOR in liver (Fig. 3f). In line with the accelerated glucose clearance, elevated expression of glucose transporter 1 (GLUT-1) and glucose transporter 2 (GLUT-2) was detected in liver of 12- to 15-month-old G3 *mTerc*^−/−^ mice compared with *mTerc*^+/+^ mice (Supplementary Fig. 1E). Glucose transporter 4 (GLUT-4) protein expression in skeletal muscle was not increased (Supplementary Fig. 1F).

*mTerc*^+/+^ mice (12- to 15-month old) showed no significant changes in blood glucose and intrahepatic glucose levels when exposed to a glucose-enriched diet (Fig. 2c,d). In contrast, feeding of the glucose-enriched diet reverted almost all starvation parameters in 12- to 15-month-old G3 *mTerc*^−/−^ mice exhibiting weight loss on normal diet. Specifically, in the liver, glucose-enriched diet normalized IGFBP-1 expression (Supplementary Fig. 1B), intracellular glucose levels (Fig. 2d), fat content (Fig. 3d) and levels of phosphorylated mTOR (Fig. 3f). In blood serum, glucose-enriched diet rescued plasma levels of glucose (Fig. 2c), GH and IGF-1 (Fig. 2b), and essential amino acids (Fig. 3e). Since the glucose-enriched diet did not contain elevated levels of essential amino acids, these data indicated that the glucose-mediated rescue of a catabolic metabolism in G3 *mTerc*^*−/−*^ mice led to the recovery of essential amino acid plasma levels.

The analysis of body weight curves showed that glucose substitution was sufficient to increase body weights of ageing G3 *mTerc*^−/−^ mice exhibiting weight loss and premature ageing (Fig. 1a). A maximum rescue occurred 4 weeks after glucose supplementation followed by a plateau before the reappearance of progressive weight loss and premature ageing. These data suggested that glucose substitution only achieved a transient rescue in tissue maintenance of telomere dysfunctional mice. In agreement with this interpretation, G3 *mTerc*^−/−^ mice that were rescued with a glucose-enriched diet after the first appearance of body weight loss redeveloped a moribund phenotype and body weight loss 2–3 months after initiating the glucose rescue (Fig. 1a,b). Glucose-fed G3 *mTerc*^−/−^ mice at the end stage of the rescue period exhibited a strong reappearance of starvation phenotypes such as a reduction in hepatic glycogen levels (Supplementary Fig. 1D) and abnormally improved glucose tolerance (Fig. 3b). A possible explanation for the transient nature of the glucose-mediated rescue was that alterations in energy metabolism in response to telomere dysfunction progressively increased the need of glucose substitution, which at some point could not be covered by the glucose-enriched diet.

### Elevated rate of glucose metabolism in ageing G3 *mTerc* KO mice

**Figure 4:**
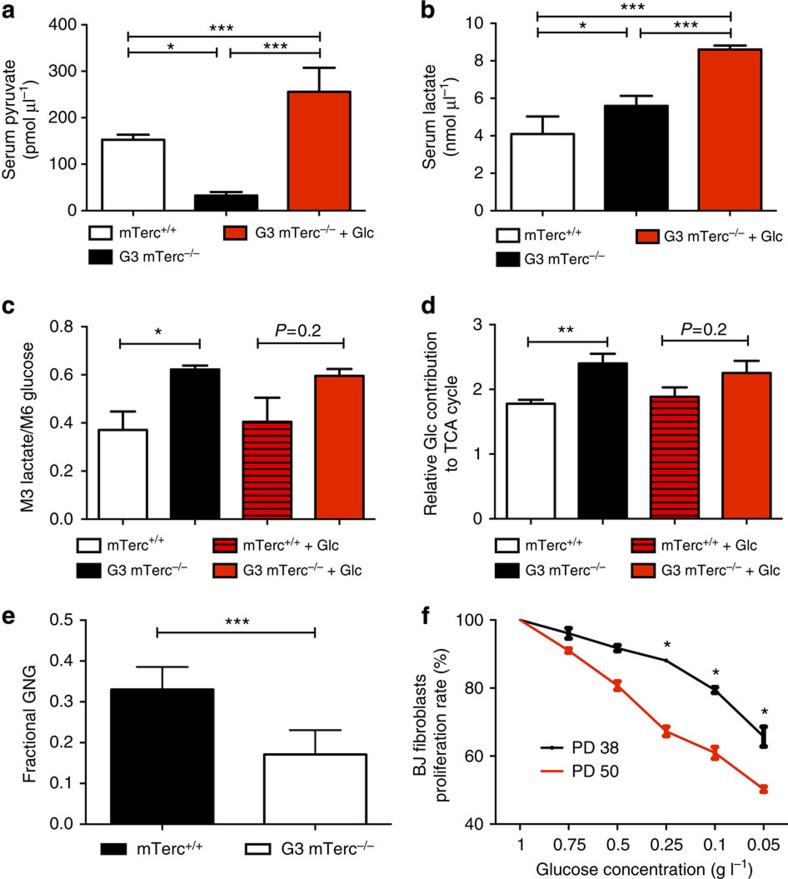
Glucose supplementation improves energy homeostasis of telomere dysfunctional mice by increasing glycolysis and oxidative glucose metabolism. 12–15 month old G3 *mTerc*^−/−^ mice with weight loss on normal ad libitum diet and age-matched *mTerc*^+/+^ mice were analyzed under continuous exposure to normal ad libitum diet or 2 weeks after switching to a glucose enriched diet (+Glc): (**a**,**b**) The bar graphs show serum levels of pyruvate (in pmol μl^−1^) (**a**) and lactate (in nmol μl^−1^) (**b**) in the indicated groups (*n*=4–6 mice per group). (**c**) Lactate production. For mice on normal diet, the amount of plasma M3 lactate isotopologues 20 min after injection of U^13^C_6_ glucose normalized by the amount of plasma M6 glucose after 10 min (*n*=4–5 mice per group). For the group on the glucose-enriched diet, two times the amount of plasma M2 lactate isotopologues 20 min after injection of 1,2^13^C_2_ glucose normalized by the amount of plasma M2 glucose after 10 min (*n*=4–5 mice per group). (**d**) The bar graph shows the ratio of 1-M0 citrate and M3-labelled lactate in liver tissue. Higher levels indicate increased relative flux into the TCA cycle (*n*= 4–7 mice per group). (**e**) The graph shows differences in the fractional gluconeogenesis (GNG) of the respective groups. Higher fractional GNG means a relative higher contribution of GNG to glucose flux (*n*=6 mice per group). (**f**) Aged human fibroblasts show a higher dependency on glucose. The graph shows the proliferation rate of BJ fibroblasts in early (PD 38) and late passage (PD 50) under different glucose concentrations (in g l^−1^) of the medium. All statistical data were assessed using Student’s *t*-test and are presented as mean±s.e.m.*P<0.05, **P<0.01, ***P<0.001.

G3 *mTerc*^−/−^ mice (12- to 15-month old) with reduced weight on normal diet exhibited a reduction in serum pyruvate levels (Fig. 4a) and increased serum lactate levels (Fig. 4b). After injection of ^13^C-labelled glucose, G3 *mTerc*^−/−^ mice showed increased levels of lactate production compared with *mTerc*^+/+^ mice (Fig. 4c). Furthermore, the flux of labelled glucose into citrate, an intermediate of the TCA cycle, was significantly elevated in liver tissue of aged mice with telomere dysfunction compared with the wild-type controls (Fig. 4d). Together, these data indicated that G3 *mTerc*^−/−^ mice with weight loss used elevated rates of glycolysis and glucose oxidation in the TCA cycle for energy production. However, the low serum pyruvate concentration in G3 *mTerc*^−/−^ mice on normal diet suggested that substrate levels became limiting under these conditions. In agreement with this interpretation, analysis of plasma glucose flux after labelled glucose injection revealed a suppression of fractional gluconeogenesis (GNG; the fraction of GNG contributing to the total glucose flux in plasma by recycling of three carbon lactate bodies back to glucose) in G3 *mTerc*^−/−^ mice on normal diet (Fig. 4e and Supplementary Fig. 2A).

When 12- to 15-month-old G3 *mTerc*^−/−^ mice that exhibited weight loss on normal diet were exposed to a glucose-enriched diet, serum pyruvate increased to extraordinary high levels within 2 weeks (Fig. 4a). Experiments with labelled glucose revealed that the relative flux of glucose into lactate as well as flux into the TCA cycle remained elevated in these G3 *mTerc*^*−/−*^ mice (2 weeks after initiation of the glucose-enriched diet; Fig. 4c,d). These data indicated that ageing telomere dysfunctional mice use elevated rates of glucose metabolism in glycolysis and in the TCA cycle. This results in an increased dependency of G3 *mTerc*^*−/−*^ mice on glucose substitution compared with *mTerc*^+/+^ controls. Under normal diet, substrates for energy generation become limiting in G3 *mTerc*^*−/−*^ mice but a glucose-enriched diet rescues substrate levels facilitating enhanced rates of glucose metabolism for maintenance of energy homeostasis in aged G3 *mTerc*^*−/−*^ mice.

To test whether telomere shortening would also affect the glucose dependency of primary human cells, experiments were conducted on BJ fibroblasts. Telomere shortening limits the proliferative capacity of human fibroblasts to 50–70 cell divisions. Early-passage fibroblasts (PD 38) and late-passage fibroblasts (PD 50) were exposed to a medium containing low levels of glutamine (250 μM). When these cells were exposed to decreasing concentrations of glucose, late-passage fibroblasts showed significantly enhanced impairments in proliferation compared with cells of the early passage (Fig. 4f). These findings indicate that under condition of low glutamine substitution, late-passage human fibroblasts with short telomeres depend more on glucose substitution to maintain proliferation rates compared with early-passage fibroblasts with long telomere reserves.

### Glucose substitution rescues mitochondrial biogenesis

**Figure 5:**
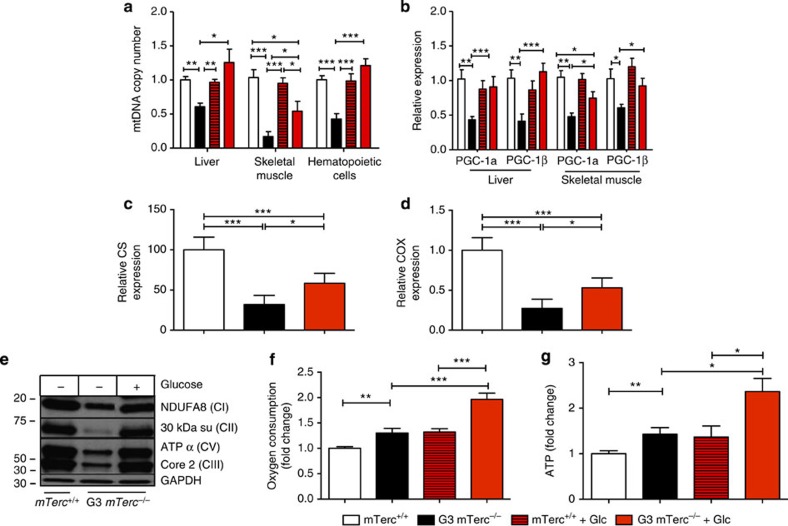
Glucose supplementation rescues mitochondrial mass and increases oxygen consumption and ATP levels in G3 *mTerc^−/−^* mice. 12–15 month old G3 *mTerc*^−/−^ mice with weight loss on normal ad libitum diet and age-matched *mTerc*^+/+^ mice were analyzed under continuous exposure to normal ad libitum diet or 2 weeks after switching to a glucose enriched diet (+Glc): (**a**–**d**,**g**,**f**) Histograms showing (**a**) the mitochondrial DNA (mtDNA) copy number in the indicated tissues, (**b**) the expression of PGC-1α and PGC-1β in the indicated tissues, (**c**) hepatic citrate synthase (CS) expression, (**d**) hepatic cytochrome c oxidase levels (COX), (**f**) oxygen consumption of freshly isolated haemtopoietic cells (Lin-negative), and (**g**) ATP-levels of freshly isolated haemtopoietic cells (Lin-negative). Data in all histograms are shown as relative expression levels/numbers with data for *mTerc*^+/+^ mice on normal ad libitum diet being set to 1. (**a**-**d**,**g**) *n*=4–6 mice per group, (**f**) *n*=9–17 mice per group. (**e**) Representative western blot analysis of OXPHOS enzymes ((NDUFA (CI), 30 kDa su (CII), ATPα (CV), Core 2 (CIII)) in liver homogenates. All statistical data were assessed using Student’s *t*-test and are presented as mean±s.e.m. *P<0.05, **P<0.01, ***P<0.001.

Previous studies revealed that telomere dysfunction leads to impairments in mitochondrial biogenesis and function by activating p53/p21-dependent signalling^6^,^7^. In agreement with these studies, 12- to 15-month-old G3 *mTerc*^−/−^ mice that lost body weight on normal diet exhibited impaired mitochondrial biogenesis in various tissues (such as liver, skeletal muscle and haematopoietic cells) as indicated by reduction in mitochondrial DNA (mtDNA) copy number (Fig. 5a and Supplementary Fig. 2B) and reduced expression of transcriptional regulators of mitochondrial biogenesis (Fig. 5b). In line with reductions in mtDNA, the expression of mitochondrial enzymes (cytochrome *c* oxidase and citrate synthase) were reduced in liver lysates of 12- to 15-month-old G3 *mTerc*^*−/−*^ mice with weight loss on normal diet compared with *mTerc*^*+/+*^ mice (Fig. 5c,d).

Switching 12- to 15-month-old G3 *mTerc*^*−/−*^ mice with weight loss on normal diet to a glucose-enriched diet significantly rescued mitochondrial biogenesis in various tissues as indicated by increases in mtDNA copy numbers (Fig. 5a), the expression of transcriptional regulators of mitochondrial biogenesis (Fig. 5b), mitochondrial enzymes (Fig. 5c,d) and mitochondrial complexes I, II, III and V (Fig. 5e). Of note, glucose-mediated improvements in mitochondrial biogenesis were incomplete in skeletal muscle (Fig. 5a,b). Together, these data indicated that telomere dysfunction induces impairments in mitochondrial biogenesis but these defects were rescued by glucose substitution, especially in liver and partially in skeletal muscle.

To assess mitochondrial respiratory activity, freshly extracted bone marrow cells (Lin-negative) were analysed for oxygen consumption. Although exhibiting significant reduction in mitochondrial copy number (Fig. 5a), haematopoietic cells from aged telomere dysfunctional mice on normal diet showed significantly higher basal rates of oxygen consumption and ATP levels compared with *mTerc*^*+/+*^ mice (Fig. 5f,g). Glucose substitution with subsequent increase of the mtDNA copy number (Fig. 5a) led to a further strong increase in oxygen consumption and ATP levels of freshly isolated haematopoietic cells from 12- to 15-month-old G3 *mTerc*^*−/−*^ mice compared with all other groups (Fig. 5f,g). Together, these results indicated that cells from aged mice with dysfunctional telomeres contain reduced numbers of mitochondria, but these mitochondria were hyperactive receiving an elevated flux of glucose-derived carbon and show elevated rates of oxygen consumption compared with *mTerc*^*+*/+^ mice. Increase in ATP levels in response to glucose feeding and stimulation of mitochondrial biogenesis in glucose-fed, 12- to 15-month-old G3 *mTerc*^*−/−*^ mice indicated that mitochondria were functional, which was also supported by the normal morphology of mitochondria of G3 *mTerc*^*−/−*^ mice in electron microscopy (Supplementary Fig. 2C). Together, these results showed that haematopoietic cells of G3 *mTerc*^*−/−*^ mice exhibit an elevated ATP production and increased glucose degradation via glycolysis and oxidative metabolism in the TCA cycle. Under normal diet, glucose levels were not sufficient to cover this elevated demand for energy production resulting in a catabolic phenotype (Fig. 3) and a starvation response (Figs 1 and 2) in ageing G3 *mTerc*^*−/−*^ mice.

**Figure 6:**
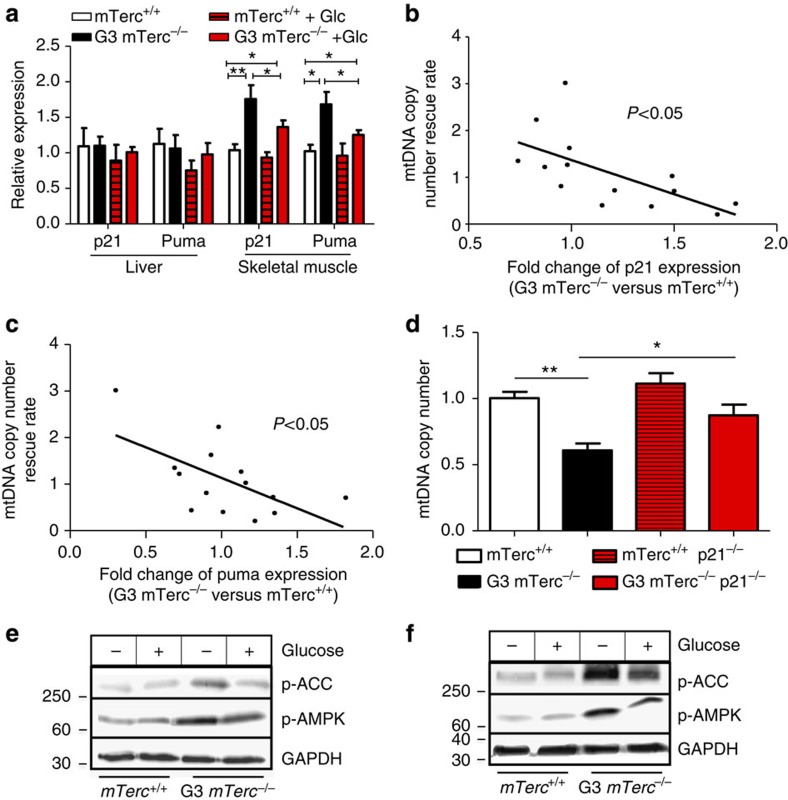
DNA damage signalling impairs mitochondrial biogenesis and function in ageing G3 *mTerc*^*−/−*^ mice. 12–15 month old G3 *mTerc*^−/−^ mice with weight loss on normal ad libitum diet and age-matched *mTerc*^+/+^ mice were analyzed under continuous exposure to normal ad libitum diet or 2 weeks after switching to a glucose enriched diet (+Glc): (**a**) Relative expression of the DNA damage signalling members (p21 and PUMA) in liver and skeletal muscle (*n*=4–5 mice per group). Activation of p53 targets occurs in skeletal muscle but not in liver of G3 *mTerc*^*−/−*^ mice. Glucose supplementation partially rescued p53 activation amplified in skeletal muscle of G3 *mTerc*^*−/−*^ mice, suggesting that starvation responses in G3 *mTerc*^*−/−*^ mice with weight loss on normal diet could amplify the activation of p53 in tissues. Data for *mTerc*^*+/+*^ mice were set to 1. (**b**,**c**) There is a direct relation between the activity of DNA damage signalling and mitochondrial biogenesis induced by glucose supplementation. The scatter plots show the relation between the mitochondrial DNA (mtDNA) copy number rescue rate and the relative expression of p21 (**b**) and PUMA (**c**) in both muscle and liver tissue of ageing G3 *mTerc*^*−*/−^ mice. The mtDNA copy number rescue rate was calculated by dividing the delta in mtDNA copy numbers in G3 *mTerc*^*−*/−^ mice on glucose-enriched diet compared with G3 *mTerc*^*−*/−^ mice on normal diet through the delta in mtDNA copy numbers in G3 *mTerc*^*−*/−^ mice on normal diet compared with wild-type mice on normal diet (*n*=7 mice per group). (**d**) KO of p53 downstream targets rescue impaired mitochondrial biogenesis in G3 *mTerc*^*−/−*^ mice. The bar graph shows relative mtDNA copy number in liver of 12- to 15-month-old G3 *mTerc*^*−/−*^ mice and controls, as well as age-matched G3 *mTerc*^*−/−*^ mice, p21^−/−^ double KO mice and controls (*n*=4–5 mice per group). Data for *mTerc*^*+/+*^ mice were set to 1. (**e**,**f**) Western blot analysis of protein levels of phosphorylated ACC (p-ACC; as a phosphorylated AMPK (p-AMPK; target) and phosphorylated AMPK in frozen liver (**e**) and skeletal muscle (**f**) extracts of the indicated groups (*n*=4–6 mice per group). All statistical data were assessed using Student’s *t*-test and are presented as mean±s.e.m. *P<0.05, **P<0.01.

To understand mechanisms of improved mitochondrial biogenesis in glucose-fed G3 *mTerc*^−/−^ mice, upstream regulators of mitochondrial biogenesis were analysed including p53, IGF-1 and mTOR signalling. Telomere dysfunction induces p53, which in turn can impair mitochondrial biogenesis by suppressing PGC1-α and PGC1-β signalling^6^. The analysis of p53 target genes (PUMA and p21 mRNA expression) revealed heterogeneity in p53 activation in liver and skeletal muscles of aged G3 *mTerc*^−/−^ mice with weight loss on normal diet compared with control cohorts. Specifically, there was no induction of p53 targets in liver but a significant induction in skeletal muscle of aged G3 *mTerc*^−/−^ mice with weight loss on normal diet compared with age-matched wild-type mice (Fig. 6a). Comparing levels of p53 activation with the level of glucose-mediated improvements in mitochondrial biogenesis, 2 weeks after the diet change in individual tissue samples of aged G3 *mTerc*^*−*/−^ mice, revealed a negative correlation between both parameters (Fig. 6b,c). Similar results were obtained in an analysis focusing on p53 expression and mitochondrial biogenesis only in the skeletal muscle tissue (Supplementary Fig. 3A,B). Specifically, tissue samples with elevated p53 activity exhibited weaker improvements in mitochondrial copy numbers after glucose supplementation compared with tissue samples with low p53 activation. These results indicated that both, p53-dependent mechanisms and glucose deficiency *per se*, contributed to impairments in mitochondrial biogenesis in G3 *mTerc*^*−*/−^ mice. Thus, glucose feeding cannot completely revert defects in mitochondrial biogenesis in tissues with elevated p53 activity. In line with this interpretation, KO of p21—a downstream target of p53, which was implemented in suppression of mitochondrial function in response to telomere dysfunction^7^—also led to a rescue of mitochondrial biogenesis in aged G3 *mTerc*^*−*/−^ mice (Fig. 6d).

### Alterations in metabolic signalling pathways impair mitochondrial biogenesis

To understand glucose-dependent mechanisms that contribute to defects in mitochondrial biogenesis in ageing telomere dysfunctional mice, the activity of AMP-activated protein kinase (AMPK), IGF-1 and mTOR signalling was analysed. It was shown that AMPK-dependent activation of p53 occurs in response to glucose deprivation^23^. These data suggested that glucose-mediated improvements in mitochondrial biogenesis could involve the amelioration of AMPK-dependent p53 activation in ageing G3 *mTerc*^*−/−*^ mice. There was a strong induction of AMPK (and its target phosphorylated acetyl-CoA carboxylase (ACC)) in liver and muscle of 12- to 15-month-old G3 *mTerc*^−/−^ mice with weight loss on normal diet compared with *mTerc*^+/+^ mice (Fig. 6e,f and Supplementary Fig. 3C–F). The glucose-enriched food partially reduced AMPK activation in both compartments of G3 *mTerc*^−/−^ mice (Fig. 6e,f and Supplementary Fig. 3C–F). Together, these data suggested that glucose-mediated reduction in AMPK activation could contribute to reduction in p53 activation and improvements in mitochondrial biogenesis in skeletal muscle. However, in the liver, this pathway cannot explain glucose-mediated improvements in mitochondrial biogenesis given the lack of p53 activation in this compartment of aged G3 *mTerc*^−/−^ mice from our cohort (Fig. 6a).

**Figure 7:**
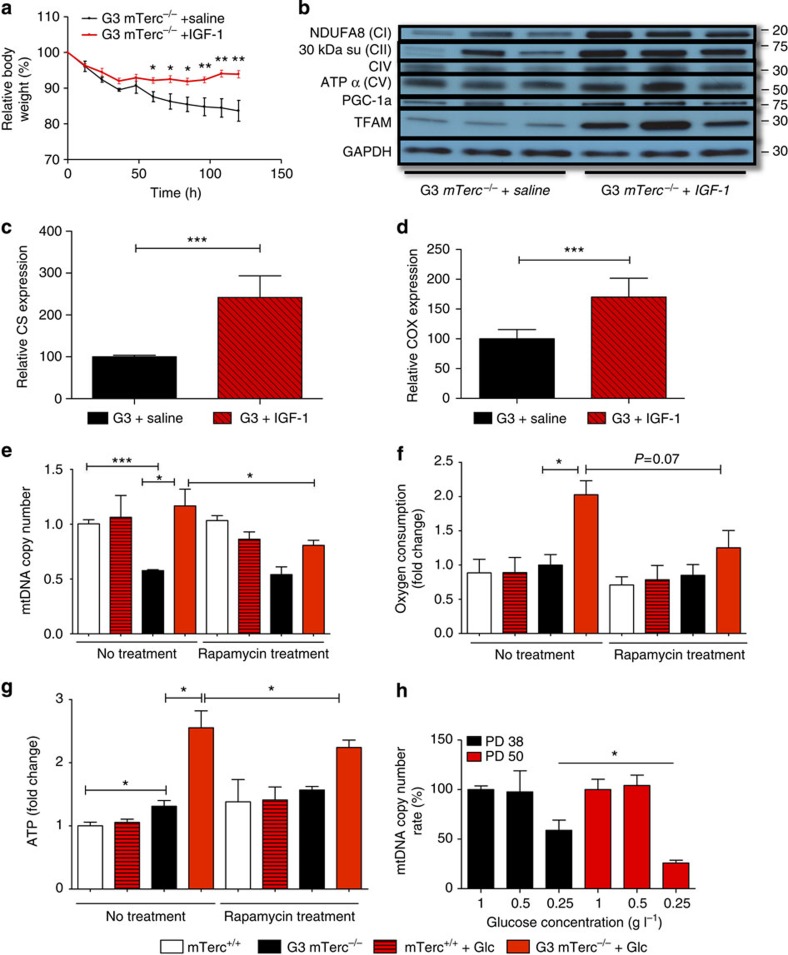
Suppression of IGF-1 and mTOR contributes to impairments in mitochondrial biogenesis and function in ageing G3 *mTerc*^*−/−*^ mice. (**a**–**d**) G3 *mTerc*^−/−^ mice (12- to 15-month old) on glucose-enriched diet for 2 weeks were shifted to normal diet and separated into two groups: one saline-treated group (*n*=4 mice) and one IGF-1-treated group (*n*=5 mice). (**a**) The graph shows the relative body weight loss of the respective groups at the indicated time points. (**b**) Western blot analysis of the respective groups for members of the respiratory chain (NDUFA (CI), 30 kDa su (CII), CIV, ATPα (CV)) as well as PGC1α and TFAM. (**c**,**d**) The bar graphs show the relative mitochondrial citrate synthase (CS) (**c**) and cytochrome *c* oxidase (COX) expression (**d**) in liver homogenates of mice with or without IGF-1 treatment. (**e**) The bar graphs show the relative mitochondrial DNA (mtDNA) copy number in liver of 12- to 15-month-old G3 *mTerc*^−/−^ mice with weight loss as well as *mTerc*^*+/+*^controls with and without glucose supplementation and rapamycin treatment (*n*=5–6 mice per group). Data for *mTerc*^*+/+*^ mice were set to 1. (**f**) The bar graph shows the relative oxygen consumption rate (OCR) of haematopoietic cells (Lin-negative, Lin−) of the depicted mouse cohorts on the indicated diets with and without rapamycin treatment (*n*=4 mice per group). Data for *mTerc*^*+/+*^ mice were set to 1. (**g**) The bar graph shows relative ATP levels of freshly isolated haematopoietic cells (Lin−) on the different diets with and without rapamycin treatment. (*n*=4 mice per group). Data for *mTerc*^*+/+*^ mice were set to 1. (**h**) The reduction of mtDNA copy number was significantly increased in late-passage fibroblasts when compared with early passage at 0.25 g l^−1^ glucose concentration in culture medium. Data are shown as relative values with mtDNA copy number at 1gl^−1^ glucose concentration set to 1. Data for *mTerc*^*+/+*^ mice were set to 1. All statistical data were assessed using Student’s *t*-test and are presented as mean±s.e.m. *P<0.05, **P<0.01, ***P<0.001.

Lifespan extension in *C. elegans* by inhibition of insulin/IGF-1 signalling was associated with reduction in mitochondrial activity in some studies^24^,^25^, and IGF-1 was shown to promote mitochondrial biogenesis in cooperation with mitogen stimulation in mammalian Schwann cells^19^. In addition, IGF-1 can prevent mitochondrial autophagy in response to serum deprivation^26^. IGF-1 serum levels were strongly reduced in telomere dysfunctional mice on normal diet but glucose substitution completely rescued IGF-1 levels (Fig. 2b). To determine whether IGF-1 contributed to the rescue in mitochondrial biogenesis in glucose-fed, telomere dysfunctional mice, a cohort of aged G3 *mTerc*^−/−^ mice was rescued from weight loss and early death by glucose re-feeding. G3 *mTerc*^−/−^ mice with an extended lifespan were then re-exposed to normal diet. Simultaneously, a treatment with subcutaneous IGF-1 application versus vehicle control was initiated. As noted before, G3 *mTerc*^−/−^ mice were highly dependent on glucose substitution and developed a rapid weight loss and a moribund phenotype within 7 days after re-exposure to normal diet (Fig. 7a). The cohort treated with IGF-1 injections, although not showing a rescue in lifespan (data not shown), exhibited significantly blunted weight loss (Fig. 7a). Improved weight maintenance was accompanied by higher proliferation rates in the intestinal epithelium—a highly proliferative compartment (Supplementary Fig. 4A). Impaired maintenance of high-turnover organs in telomere dysfunctional mice is induced by activation of p53-dependent apoptosis and cell cycle arrest^1^,^3^,^27^. However, IGF-1 application did not alter the activation of p53-dependent DNA damage signals in the intestinal epithelial compartment (Supplementary Fig. 4B–D). Instead, IGF-1 application rescued mitochondrial biogenesis as indicated by increases in the expression of PGC-1α and TFAM (Fig. 7b), mitochondrial enzymes citrate synthase and cytochrome *c* oxidase (Fig. 7c,d), and the mitochondrial complexes I, II, IV and V (Fig. 7b) in liver tissue.

The IGF-1-mediated rescue in mitochondrial biogenesis in liver of 12- to 15-month-old G3 *mTerc*^−/−^ mice was similar to the rescue mediated by feeding of a glucose-enriched diet (Supplementary Fig. 4E,F). In contrast to the glucose rescue of G3 *mTerc*^−/−^ mice, IGF-1 application did not result in a rescue of pyruvate serum levels (Supplementary Fig. 4G) and serum lactate levels did not increase (Supplementary Fig. 4H). A possible explanation for these findings indicates that G3 *mTerc*^−/−^ mice continue to depend on increased glucose substitution to maintain energy homeostasis via elevated glucose metabolism in both glycolysis and TCA cycle activity. Since IGF-1 treatment did not improve substrate levels (glucose availability and pyruvate levels), this treatment could not restore energy homeostasis although improving mitochondrial biogenesis.

In addition to direct effects on mitochondrial biogenesis, IGF-1 signalling may also affect mitochondrial biogenesis by altering mTOR signalling. mTOR can activate mitochondrial biogenesis^11^ and suppression of IGF-1 can suppress mTOR activity^28^, which in turn can induce mitophagy. Aged G3 *mTerc*^−/−^ mice with weight loss on normal diet exhibited a decrease in phosphorylated mTOR in liver, which was rescued to normal levels by glucose re-feeding (Fig. 3f). In agreement with the hypothesis that activation of mTOR contributed to the rescue in mitochondrial biogenesis in glucose-fed telomere dysfunctional mice, rapamycin treatment significantly diminished glucose-mediated increases in mitochondrial biogenesis (Fig. 7e). Substitution of rapamycin also reduced the increase in oxygen consumption, as well as the ATP levels in freshly isolated haematopoietic cells of telomere dysfunctional mice exposed to a glucose-enriched diet (Fig. 7f,g). These data suggested that mTOR activation is linked to the rescue in mitochondrial biogenesis and increases in oxygen consumption in response to glucose re-feeding of telomere dysfunctional mice.

**Figure 8:**
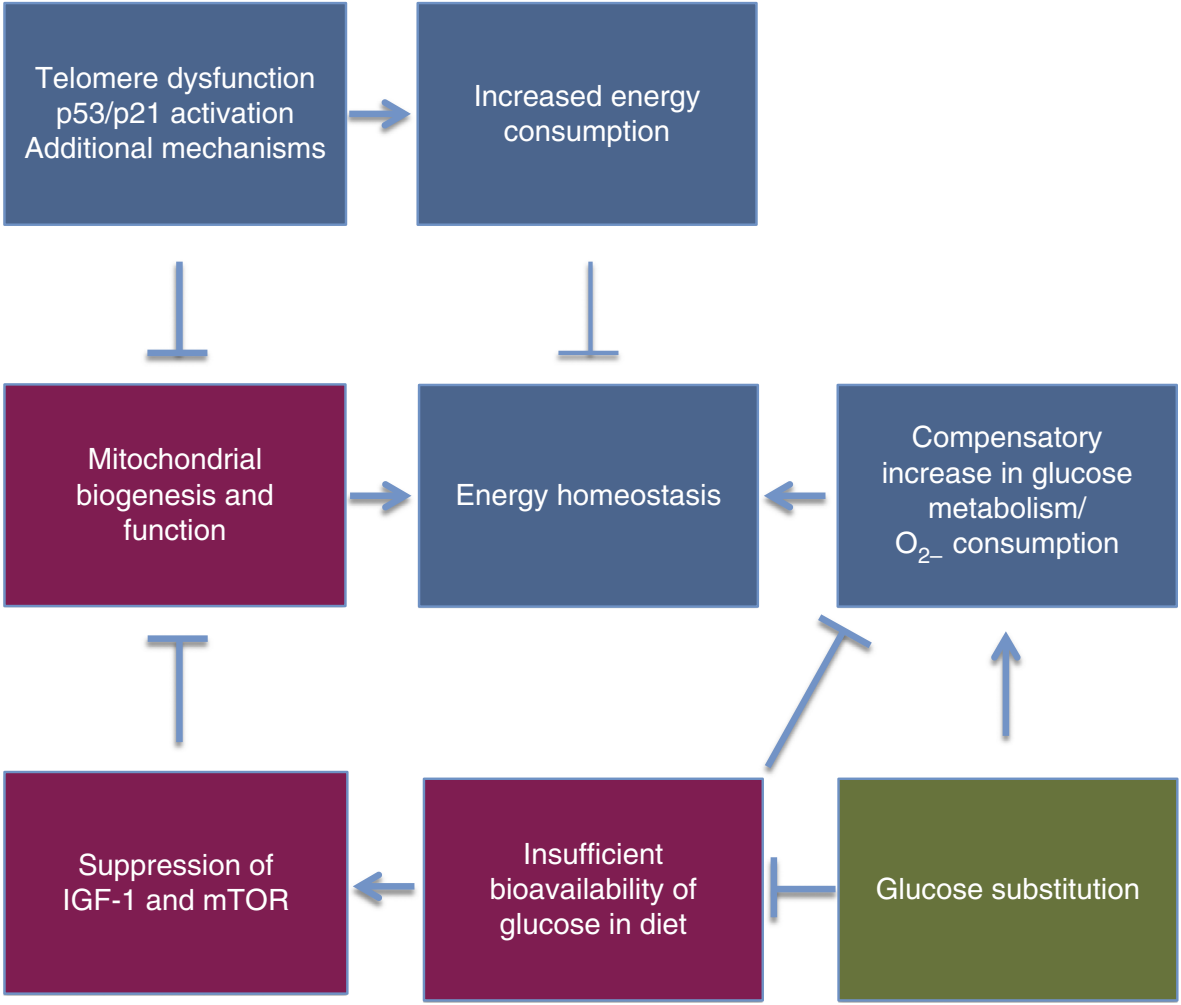
Model of telomere dysfunction-induced metabolic changes that accelerate tissue ageing. Telomere dysfunction enhances energy consumption in ageing tissues leading to compensatory increases in ATP production, glucose metabolism (via glycolysis and TCA cycle) and oxygen consumption. Under these conditions, the dietary energy content becomes limiting for the maintenance of energy homeostasis. Impairments in energy homeostasis result in catabolic changes and suppression of IGF-1/mTOR-dependent mitochondrial biogenesis, which is aggravated by the activation of p53/p21-dependent DNA damage responses in telomere dysfunctional tissues. Glucose supplementation stops this vicious circle by increasing bioavailability of substrates for glycolytic and oxidative glucose metabolism resulting in improvements of energy homeostasis and activation of IGF-1/mTOR-dependent mitochondrial biogenesis. This leads to further enhancement of oxidative glucose metabolism and ATP levels, thus prolonging the maintenance of functional tissues in the context of telomere dysfunction.

Together, these experiments revealed that p53-dependent and p53-independent processes contribute to impairments in mitochondrial biogenesis in telomere dysfunctional tissues. p53-independent processes that suppress mitochondrial biogenesis involve a deficit in energy homeostasis likely evolving as a consequence of elevated energy demand characterized by increased ATP levels, oxygen consumption and glucose metabolism in haematopoietic cells of telomere dysfunctional mice (Fig. 5f,g). In this context, energy bioavailability in the diet becomes a limiting component required for maintenance of energy homeostasis (Fig. 8). Similar metabolic changes occur in senescent human fibroblasts in response to oncogene activation^8^,^9^. To test whether human fibroblasts would also show an increased dependency on glucose substitution to maintain mitochondrial biogenesis in response to telomere shortening, mitochondrial copy numbers were analysed in early and late passages of primary human fibroblasts. Of note, the reduction in mitochondrial biogenesis in response to lowering glucose levels was significantly increased in late-passage human fibroblasts compared with early passages (Fig. 7h), suggesting that human cells also exhibit an increased dependency on glucose substitution to maintain energy homeostasis and mitochondrial biogenesis in the context of shortened telomeres.

## Discussion

The current study provides the first experimental evidence that dietary requirements of mice change during telomere dysfunction-induced ageing. Compared with wild-type mice, progeroid mice with dysfunctional telomeres show increases in energy consumption characterized by compensatory increases in ATP levels, oxygen consumption rates (OCRs) and glucose metabolism via glycolysis and the TCA cycle. In this context, normal diet does not provide sufficient glucose levels to maintain energy homeostasis resulting in catabolic metabolism (loss of fat tissue, degradation of essential amino acids and reduction in IGF-1/mTOR signalling), weight loss, impairments in organ function and premature death. Different mechanisms appear to contribute to defects in energy homeostasis in response to telomere dysfunction (Fig. 8).

*p53-dependent suppression of mitochondrial biogenesis:* in agreement with previous publications^6^,^7^, the accumulation of dysfunctional telomeres leads to p53 and p21 activation, which in turn suppresses mitochondrial biogenesis in some tissues (skeletal muscle, intestine and so on). This decrease in mitochondrial function likely contributes to the evolution of energy deficits in telomere dysfunctional tissues and an adaptive increase in the utilization of glycolysis for energy production.

*Increases in energy consumption*: despite reduction in mitochondrial copy numbers, telomere dysfunctional mice show elevated ATP levels, increased oxygen consumption and enhanced glucose metabolism in both glycolysis and TCA cycle compared with wild-type controls. Still telomere dysfunctional mice exhibit defects in energy homeostasis characterized by catabolic changes and decreases in body weight and organ maintenance, indicating that the compensatory increases in ATP production remain insufficient for organ maintenance. Of note, all these phenotypes are rescued by glucose supplementation. These findings support the conclusion that energy consumption is increased in telomere dysfunctional mice compared with wild-type mice resulting in compensatory increases in glucose metabolism to maintain energy homeostasis. Recent studies revealed that oncogene-induced senescence increases energy demand of fibroblasts by increasing proteolytic stress via the induction of pro-inflammatory signals as part of the senescence-associated secretory phenotype^9^. It was shown that G3 *mTerc*^−/−^ mice exhibit an increase in telomere dysfunction early in life correlating with the shortened lifespan of the mice. Interestingly, wild-type mice display similar increases in telomere dysfunction when reaching the maximum lifespan^29^. It remains an important question to analyse whether increased requirement of glucose substitution for the maintenance of energy homeostasis also occurs during advanced ageing of wild-type mice and more importantly in human ageing. Of note, there is evidence for increases in telomere shortening and DNA damage accumulation in multiple human tissues during ageing also affecting stem cell compartments^30^.

Importantly, this study shows that elevation in dietary glucose substitution is sufficient to prolong organismal function and survival in the context of telomere dysfunction by improving energy homeostasis via glycolysis and glucose oxidation. The beneficial effects of glucose substitution appear to be in part mediated by increases in IGF-1 and mTOR signalling. In line with this interpretation, IGF-1 application mimics glucose-mediated increases of mitochondrial copy numbers in telomere dysfunctional mice, and mTOR inhibition via rapamycin impairs glucose-mediated increases in mitochondrial copy numbers in the mice. The data suggest that IGF-1-dependent increases in mitochondrial function may also contribute to the IGF-1-mediated elongation in lifespan in other progeroid mouse strains^31^,^32^. In addition, impairments in the maintenance of mitochondrial copy number and function in response to mTOR suppression may also contribute to the outcome of calorie restriction (CR) on longevity in some recent studies. Along these lines, it was reported that CR has negative effects on lifespan in some mouse strains^33^,^34^. In addition, CR failed to increase lifespan in a recent primate study despite having positive effects on certain health parameters^35^. It is possible that negative effects of CR on energy homeostasis in ageing tissues could outcompete positive effects on health span.

The results of this study could have relevance for the treatment of elderly humans that show accumulation of DNA damage, dysfunctional telomeres, suppression of the somatotroph axis and mitochondriopathies^36^,^37^,^38^. There is evidence that 1/3–2/3 of geriatric patients are malnourished, and decreased body weight is directly associated with shortened survival in these patients^39^. These data indicate that defects in energy homeostasis represent a major factor limiting lifespan at advanced human age. Of note, the current study provides experimental evidence that human fibroblasts also exhibit an increased dependency on glucose substitution to maintain mitochondrial copy numbers and cell proliferation rates in the context of telomere shortening. The simplicity of glucose substitution makes it an attractive approach that should be evaluated in clinical trials for the treatment of pathophysiological conditions associated with coupled telomere/mitochondrial dysfunction.

## Methods

### Animal experiments

For the production of the *mTerc*^*−/−*^ mouse model, mice with C57BL/6J background have been used as previously described^40^. First-generation *mTerc*^*−/−*^ (G1) animals and mTerc^+/+^ controls were derived from heterozygous intercrosses (mixed genetic background). Following the mating between mice of the same generations, *mTerc*^*−/−*^ animals were produced up to the third generation (G3) were produced. For the individual experiments, mice aged 12–15 months were used. Furthermore, all animals were housed in a pathogen-free area (20–22 °C) with free access to food and water. Rescue experiments were performed on terminally ill mice that lost 20% of body weight and in aged mice without loss of weight. These were put on a regime (SNIFF, cat no. E15629-34) consisting of a diet with >50% glucose content. All mouse experiments were approved by the State Government of Baden-Württemberg.

### Cell culture

BJ fibroblasts at young and old passage (PD 38 and PD 50) were seeded into six-well plates for 50,000 fibroblasts per well and cultured with DMEM medium (Sigma, D5030) with 250 μmol l^−1^ glutamine and different concentrations of glucose (0.05, 0.1, 0.25, 0.5, 0.75 and 1 g l^−1^). The cells were cultured at 5% CO_2_, were incubated at 37 °C for 9 days and proliferation rate was measured with crystal violet method.

### Immunoblot analyses

Whole-cell extracts were prepared according to standard protocols and tested by western blot using anti-GAPDH (Bethyl; dilution: 1:25,000), anti-IGFBP-1 (Santa Cruz; dilution:1:1,000), anti-phospho acetyl-coA-carboxylase (Cell Signaling; dilution: 1:2,000), anti-phospho-AMPK (Cell Signaling; dilution: 1:2,000), anti-phospho-mTOR (Cell Signaling; dilution: 1:2,000), anti-phospho-p53 (Cell Signaling; dilution: 1:2,000), anti-p21 (Santa Cruz; dilution: 1:1,000), anti-gH2Ax (Millipore; dilution: 1:1,000), anti-β-actin (Sigma; dilution: 1:10,000), anti-PKM2 (Santa Cruz; dilution: 1:1,000), anti-PDHK1 (Cell Signaling; dilution: 1:2,000), OxPhos Complex Kit (Anti-Rt/ms; Invitrogen), anti-TFAM (Calbiochem; dilution: 1:1,000) and anti-PGC1α (Abcam; dilution: 1:1,000).

### Plasma measurements

Plasma levels of IGF-1 (Mediagnost), GH (Millipore), and pyruvate and lactate (Abcam) were measured by corresponding enzyme-linked immunosorbent assays.

### Glucose and insulin tolerance test

Glucose tolerance was performed after 16 h fasting by injecting intraperitoneally (i.p.) glucose (2 g kg^−1^ of body weight). For insulin tolerance test, mice were starved for 2 h followed by an injection of 0.75 U kg^−1^ of body weight insulin i.p.. Glucose levels were measured from tail blood at indicated time points using a glucometer (One Touch Ultra).

### Glycogen determination

Glycogen was measured as previously described^41^.

### RNA extraction and real-time PCR

RNA was isolated from liver using TRIZOL (Life Technologies) and cDNA was prepared using SuperScript III Reverse Transcriptase (Invitrogen). Synthesized cDNA was used for real-time PCR using fluorescent dye (SYBR Green, Qiagen). The results were normalized for comparison by measuring β-actin mRNA levels in each sample. Primers used for real-time PCR are described in the Supplementary Table 2.

### mtDNA copy number

mtDNA copy number is measured as previously described^6^. Briefly, liver and heart tissues were digested with 0.1 mg ml^−1^ Proteinase K in digestion buffer (100 mM NaCl, 10 mM Tris–Cl (pH 8.0), 25 mM EDTA, (pH 8.0) and 0.5% (w/v) SDS) at 50 °C overnight and genomic DNA are isolated with phenol/chloroform method. Real time quantitative polymerase chain reaction (RT-qPCR)-based mitochondrial quantification was performed with two different primer sets for genomic and mitochondrial loci. RT-qPCR was analysed by ΔΔ*C*_t_ method.

### Immunohistochemistry

Immunofluorescence was performed in paraffin-embedded livers using primary antibodies for proliferating cell nuclear antigen (PCNA) (Santa Cruz; dilution: 1:150), p53 (Calbiochem; dilution: 1:150) and p21 (Santa Cruz; dilution: 1:150).

### Oil-red-O staining

An amount of 5 μM frozen liver sections were fixed in 10% ice-cold formalin, washed with absolute propylene glycol and stained with 0.5% Oil-red-O solution. Stained slides were washed with 85% propylene glycol solution and rinsed with water. The slides were finally stained with haematoxylin and analysed by microscopy.

### Metabolite measurements

The targeted metabolomics approach was based on the measurements with the Absolute*IDQ* p150 kit (Biocrates Life Sciences AG, Innsbruck, Austria) allowing simultaneous quantification of 163 metabolites. The metabolomics data set includes 14 amino acids, which concentrations are reported in μM.

### Isolation of mitochondria and measurement of respiratory chain (RC)-complex activity

Liver homogenates were prepared by homogenizing a snap-frozen liver piece (50 mg) in 500 μl of 10 mM HEPES (pH 7.4), 0.5 mMEDTA, 0.5 m MEGTA and 250 mM sucrose, and were used immediately. Enzyme activities were determined spectrophotometrically as described^42^. Protein concentrations were estimated by the method of Bradford using bovine serum albumin as a standard.

### IGF-1 injection

Aged telomere dysfunctional mice losing weight were rescued with high-glucose diet. After stabilization of body weight gain mice were shifted to regular diet and divided into saline solution and IGF-1-injected mice at a dose 0.2 μg g^−1^ of body weight every 12 h.

### Rapamycin treatment

Mice were administered daily with 10 mg kg^−1^ of rapamycin (LC Laboratories, R-5,000) by i.p. injection for a period of 2 weeks. Rapamycin was first dissolved in ethanol to 50 mg ml^−1^ and then diluted 1/50 to 1 mg ml^−1^ with Ringer’s solution (containing 5.2% polyethylene glycol (PEG) and 5.2% Tween 80).

### Energy absorption and calorimetry

Mouse faeces were collected from each cage every 24 h for 5 days parallel to the measurement of food intake. All faeces were weighed and dried at 60 °C for 1 week in a standard oven. The gross energy content of the faeces was measured using adiabatic bomb calorimetry (Gallenkamp CBA-305, Loughborough, UK). Samples were run in triplicate.

### Extraction of plasma samples

All plasma samples were aliquoted at 5 μl in 1.5 ml Eppendorf tubes and stored at −80 °C until analysis. For liquid extraction, 45 μl of an ice-cold methanol/H_2_O mixture (8:1 v/v) was added to the 5 μl of plasma sample. The mixture was vortexed for 10 s and shaked at maximum speed on a thermomixer (Eppendorf) for 5 min at 4 °C. After incubation, the mixture was centrifuged for 5 min at 4 °C at 19,600 *g*, and 30 μl of the supernatant was transferred into a gas chromatography/mass spectrometry (GC/MS) vial for speed vacuum evaporation at −4 °C using a refrigerated CentriVap concentrator (Labconco). Dried samples were submitted to GC/MS analysis.

### Extraction of tissue samples

*Pulverization*. All tissue samples were weighed, collected in Eppendorf tubes and stored at −80 °C until analysis. On the day of the analysis, three 7-mm grinding balls were added to each tissue sample into the Eppendorf tube. The sample tubes were then put in liquid nitrogen for 15 s. At the same time point, the grinding block was frozen in liquid nitrogen for 15 s. Sample tubes were then put into the grinding block and milled in the ball mill (Retsch) for 2 min at 25 s^−1^ to yield a fine powder.

*Homogenization*. For homogenization, five small grinding balls (1 mm) and the appropriate amount of extraction fluid (MeOH/H_2_O, 40/8.5 v/v) were added to the pulverized samples (485 μl per 100 mg tissue) and milled for 2 min at 25 s^−1^ leading to a homogeneous fluid.

*Extraction*. A liquid–liquid extraction method was used to extract metabolites simultaneously in each tissue type. First chloroform (400 μl per 100 mg tissue) was added to the homogenized tissue fluid followed by H_2_O (200 μl per 100 mg tissue). The mixture was incubated on a thermomixer (Eppendorf) for 20 min at 1,300 r.p.m. and 4 °C. After the incubation period, the suspension was centrifuged for 5 min at 10,000 r.p.m. and 4 °C, and 30 μl of the upper aqueous phase were transferred into a GC/MS vial for speed vacuum evaporation at −4 °C using a refrigerated CentriVap concentrator (Labconco). Dried samples were submitted to GC/MS analysis.

### GC/MS analysis

Metabolite derivatization was performed using a Gerstel Multi Purpose (MPS) autosampler. Dried polar metabolites were dissolved in 15 μl of 2% methoxyamine hydrochloride in pyridine at 40 °C. After 30 min, an equal volume of MSTFA (2,2,2-trifluoro-*N*-methyl-*N*-trimethylsilyl-acetamide)+1% TMCS (chloro-trimethyl-silane) were added and held for 30 min at 45 °C. GC/MS analysis was performed using an Agilent 7,890A GC equipped with a 30-m DB-35MS capillary column. The GC was connected to an Agilent 5,975C MS operating under electron impact ionization at 70 eV. The MS source was held at 230 °C and the quadrupole at 150 °C. The detector was operated in scan mode and 1 μl of derivatized sample was injected in splitless mode. Helium was used as carrier gas at a flow rate of 1 ml min^−1^. The GC oven temperature was held on 80 °C for 6 min and increased to 300 °C at 6 °C min^−1^. After 10 min, the temperature was increased to 325 °C at 10 °C min^−1^ for 4 min. The run time of one sample was 59 min.

### Stable-isotope labelling experiments

[U^13^C_6_] or [1,2^13^C_2_] glucose was injected i.p. (0.5 g kg^−1^ body weight (BW)). Blood was taken from the mouse tail at the respective time points. Mass isotopomer distributions were determined using the MetabolieDetector software package^43^,^44^. Chemical formulas of respective fragment ions were taken from^45^. To determine the glucose turnover in plasma, the amount of M6 glucose isotopologues in plasma was measured 180 and 10 min post injection. To compensate for variation caused by i.p. injection, the amount of M6 isotopologues 180 min post injection was normalized by the amount of M6 isotopologues measured 10 min post injection. Fractional GNG in liver was determined as described by Kelleher^46^: (M1_glucose_+M2_glucose_+M3_glucose_ )/(2 × M0_lactate_ × (M1_lactate_+M2_lactate_ +M3_lactate_)).

To determine the relative glucose contribution to the TCA cycle in the liver, the mass isotopomer distribution (MID) of citrate was measured after 60 min post injection. To compensate for variation caused by i.p. injection, the amount of isotopically enriched isotopologues ( 1−M0_citrate_) was normalized by the amount of M3 isotopologues of lactic acid: 1−M0_citrate_/M3_lactate_.

### Measurement of OCR

Seahorse XF96 was used to measure the rate change of dissolved oxygen (OCR) in the culture medium. Per well, 2.2 × 10^5^ freshly isolated bone marrow cells were seeded in unbuffered DMEM in poly-lysine treated XF96 plates 1 h before the assay and the measurement was made over 2 min according to the companies’ protocol.

## Author contributions

P.M., Y.Z., L.M.G. and G.v.F. contributed to equal parts. P.M., Y.Z., L.M.G. and G.v.F. performed most of the experiments, were involved together with K.L.R. in design and analysis of the experiments, as well as in preparation of the manuscript. A.W., T.B. and K.H. performed glucose tracing experiments. S.R.C. was involved in the IGF-1 injection experiment. A.G. was involved in the survival experiments. G.H., M.D.B. and C.G. performed Seahorse experiments. V.W. and T.W. did investigations on mitochondria. R.W.S. and T.I. performed the metabolomics experiments. Z.S. was involved in thymus experiments. S.K. did bomb calorimetry and stool analysis. B.O.B. was involved in IGF-1 experiments as well as preparation of the manuscript. K.L.R. conceived the study.

## Additional information

**How to cite this article:** Missios, P. *et al.* Glucose substitution prolongs maintenance of energy homeostasis and lifespan of telomere dysfunctional mice. *Nat. Commun.* 5:4924 doi: 10.1038/ncomms5924 (2014).

## Supplementary Material

Supplementary Information Supplementary Figures 1-5 and Supplementary Tables 1-2
