# Supplementary material for: Glucose substitution prolongs maintenance of energy homeostasis and lifespan of telomere dysfunctional mice
Source: Nat Commun. 2014 Sep 18;5:4924. doi: 10.1038/ncomms5924 (PMC4199114; doi:10.1038/ncomms5924)
Supplement: Supplementary Information — Supplementary Figures 1-5 and Supplementary Tables 1-2 [file ncomms5924-s1.pdf]

Supplementary Figure 1

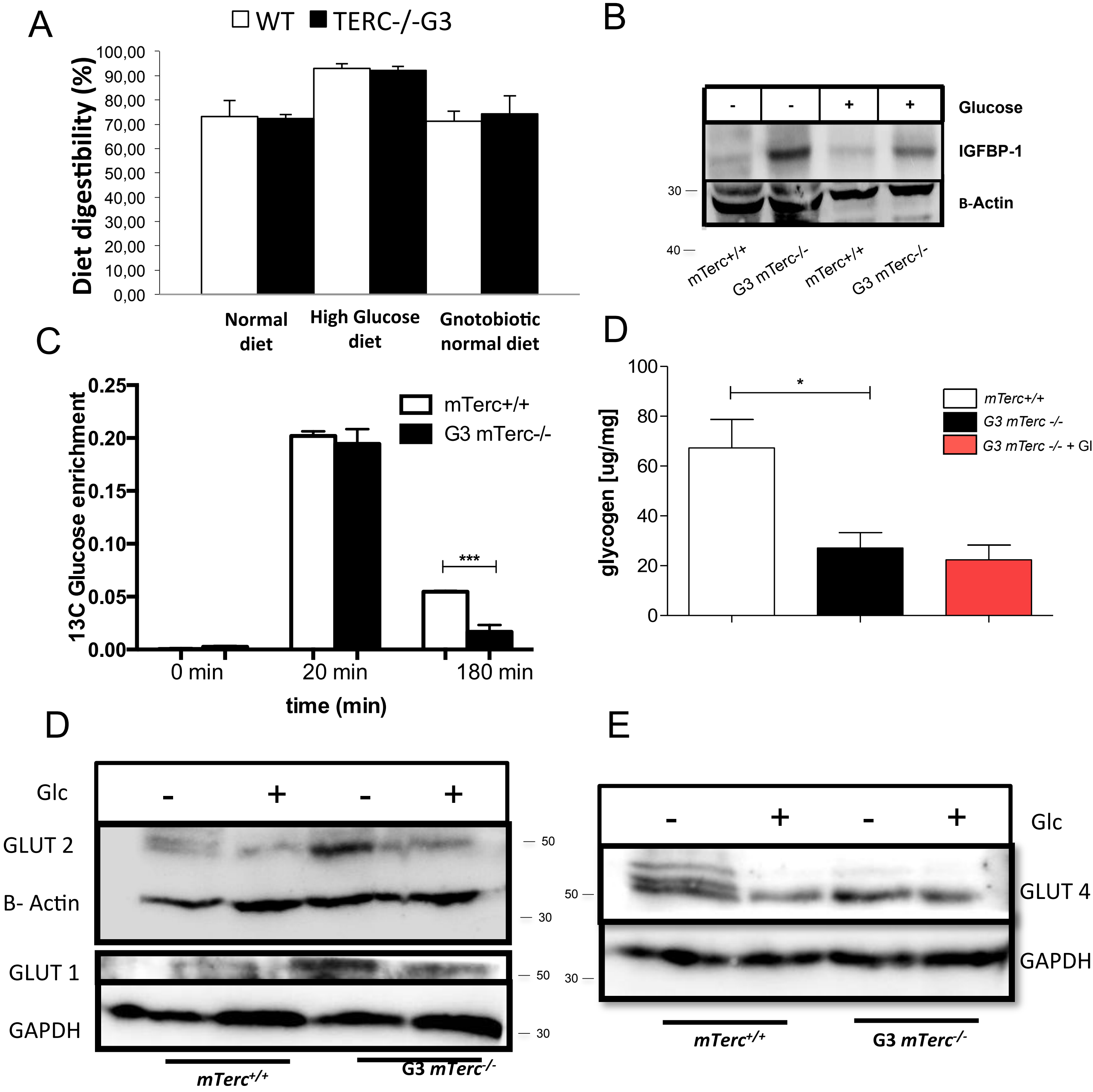

Supplementary Figure 1:

A) The bar graph shows the relation between consumed and excreted energy of the different diets by the different mice. Note that mice on high glucose consume and absorb the glucose diet more efficient than the mice on normal diet (n=9-11 mice per group). All statistical data were assessed using Student's t-test and are presented as mean±s.e.m. * = p<0.05, ** = p<0.01, *** = p<0.001.

B) Representative western blot of IGFBP-1 expression in pooled liver samples of the respective genotypes (n=4-9 per group).

C) ^{13}C labeled glucose levels in plasma after 0, 20 and 180 minutes (n=4-5 mice per group).

D) Quantification of glycogen in livers of 12-15 month old mice of the indicated genotypes and treatments (n=9-14 mice per group). Of note, these analyses were performed 2-3 months after diet change.

E,F) Western Blot of GLUT-1 and -2 **(D)** and GLUT-4 **(E)** in pooled liver **(D)** and muscle tissue **(E)** samples (n=4-5 mice per group).

All statistical data were assessed using Student's t-test and are presented as mean±s.e.m. * = p<0.05, ** = p<0.01, *** = p<0.001.

Supplementary Figure 2

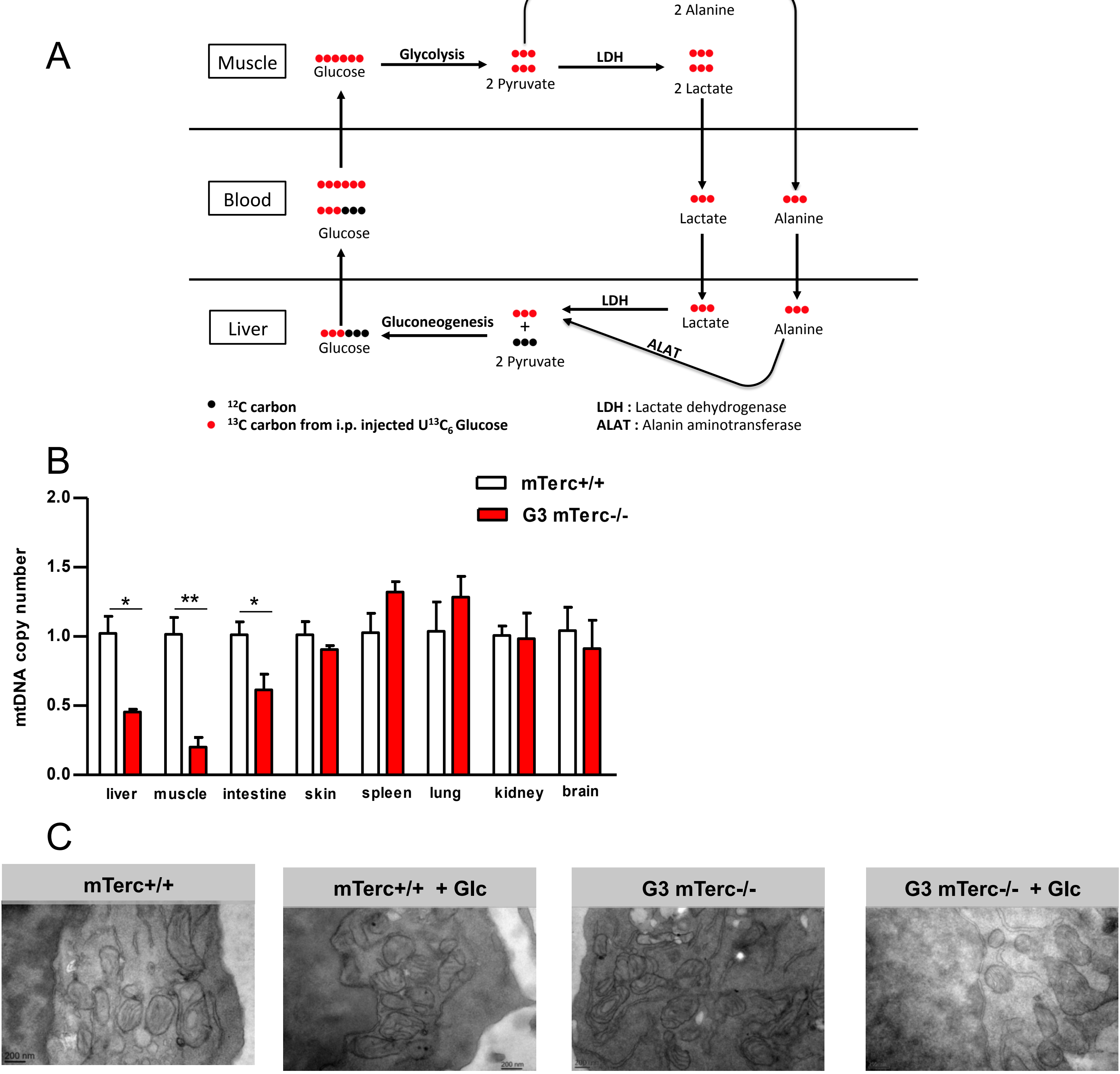

Supplementary Figure 2:

A) The figure shows a simplified version of the Cori-cycle. Muscle glucose is catabolized to pyruvate. Pyruvate is either converted to alanine by transamination or reduced to lactate. Both molecules are transported by the blood to the liver where they are converted back to pyruvate. The liver recycles the carbon of the trioses back to glucose via gluconeogenesis and finally releases the glucose back to the blood. To estimate the fractional gluconeogenesis, we injected U^{13}C$_6$ glucose (^{13}C carbon isotopes in red) into the animals. The simplified illustration depicts the flow of the tracer through the Cori cycle. Based on the observed mass isotopomer distributions (MIDs) in liver tissue and plasma we estimated the fractional gluconeogenesis according to Kelleher[41]. As the amount of isotopic enriched lactate in the liver is low, we can neglect the possibility of glucose formation by two labeled trioses.

B) Mitochondrial DNA (mtDNA) copy number in the indicated organs of 12 to 15 month old *mTerc*$^{+/+}$ and G3 *mTerc*$^{-/-}$mice (n=4-5 mice per group).

C) Electron microscopic pictures show the mitochondrial structure of bone marrow cells from 12-15 month old G3 *mTerc*$^{-/-}$ mice with weight loss and wildtype controls on normal diet as well as glucose-enriched diet (two weeks after diet-change) (n=4 mice per group). The line indicates 200nm. In conclusion, there were no structural differences observed in mitochondria of the respective genotypes.

Supplementary Figure 3

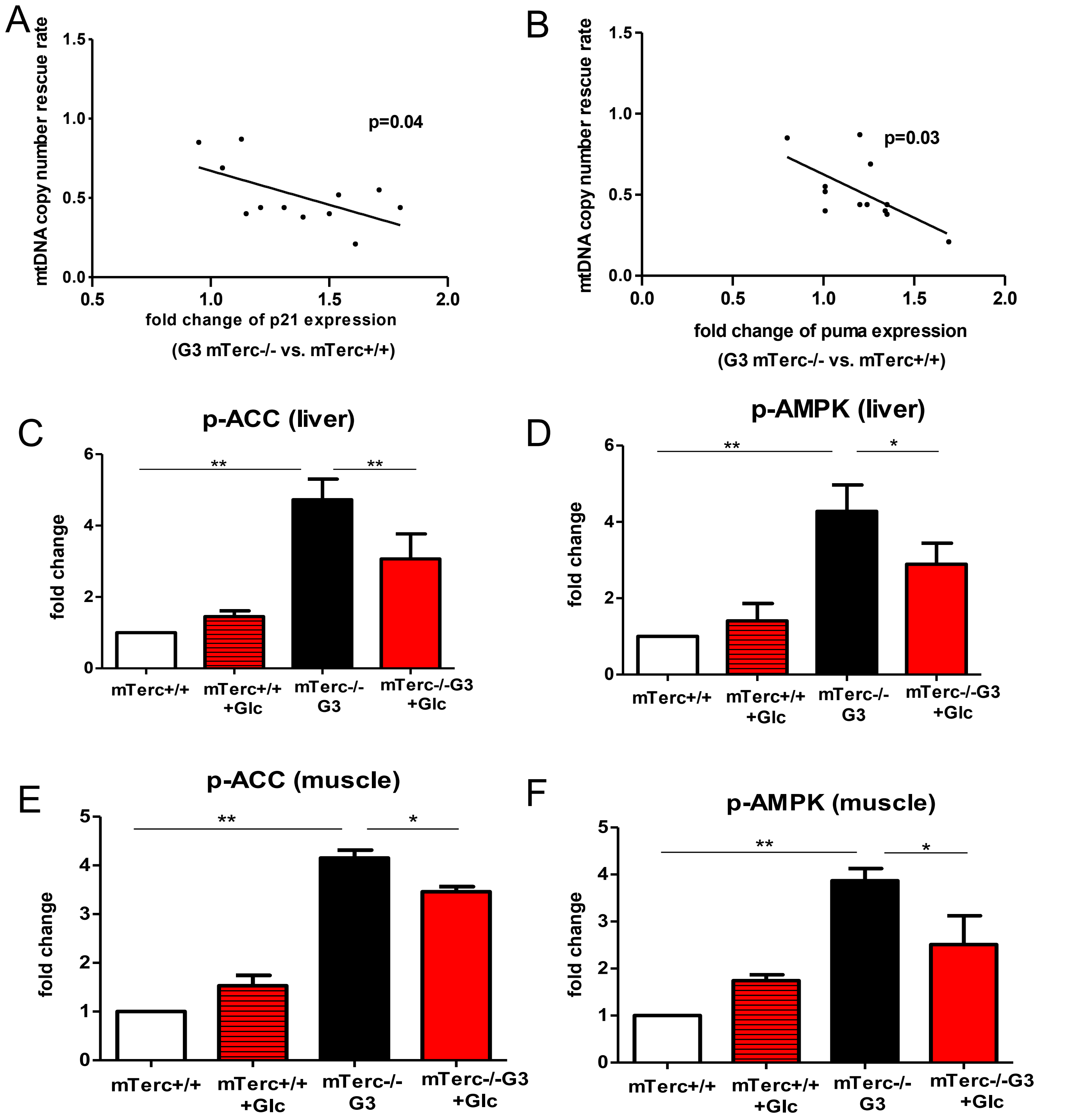

Supplementary Figure 3

A, B) There is a direct relation between the activity of DNA-damage signaling and mitochondrial biogenesis induced by glucose supplementation. The scatter plots show the relation between the mtDNA copy number rescue rate and the relative expression of p21(**A**) and PUMA (**B**) in skeletal muscle of aging G3 *mTerc*$^{-/-}$ mice. The mtDNA copy number rescue rate was calculated by dividing the delta in mtDNA copy numbers in G3 *mTerc*$^{-/-}$ mice on glucose-enriched diet compared to G3 *mTerc*$^{-/-}$ mice on normal diet through the delta in mtDNA copy numbers in G3 *mTerc*$^{-/-}$ mice on normal diet compared to wildtype mice on normal diet.
C-F) Quantification of western blot analysis of phosphorylated ACC (**C**) and phosphorylated AMPK (**D**) in whole liver homogenates as well as phosphorylated ACC (**E**) and phosphorylated AMPK (**F**) in frozen skeletal muscle sections of 10 to 15 month old mice with or without glucose supplementation of the respective groups. Western blots were run in pooled samples from the indicated mice (n= 4-6 mice per pool). ImageJ Software was used for brightness quantification. Calculated levels for p-ACC and p-AMPK were normalized by GAPDH loading control. Data for *mTerc*$^{+/+}$ mice were set to 1.
All statistical data were assessed using Student's t-test and are presented as mean±s.e.m.* = $p<0.05$, ** = $p<0.01$, ***= $p<0.001$.

Supplementary Figure 4

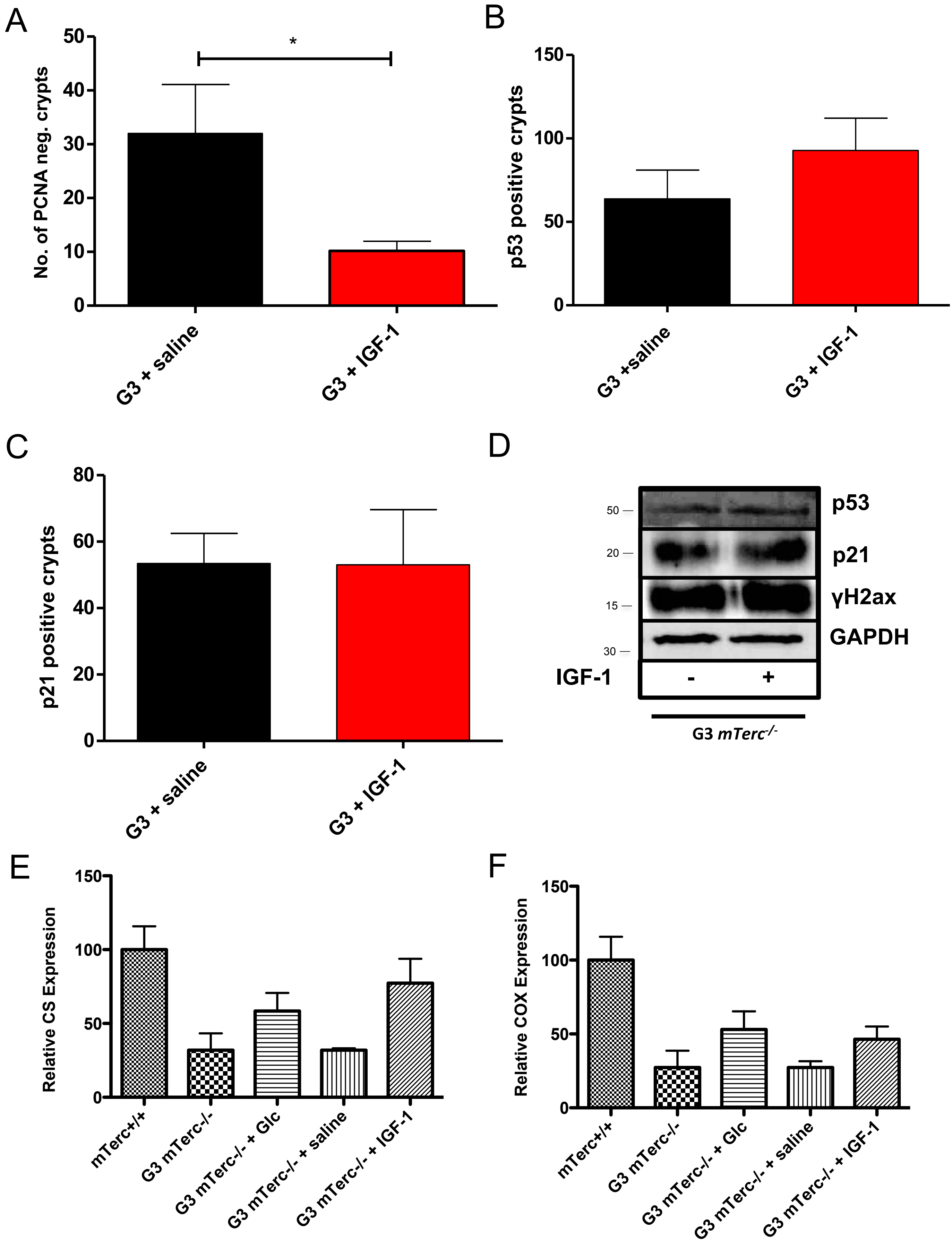

Supplementary Figure 4

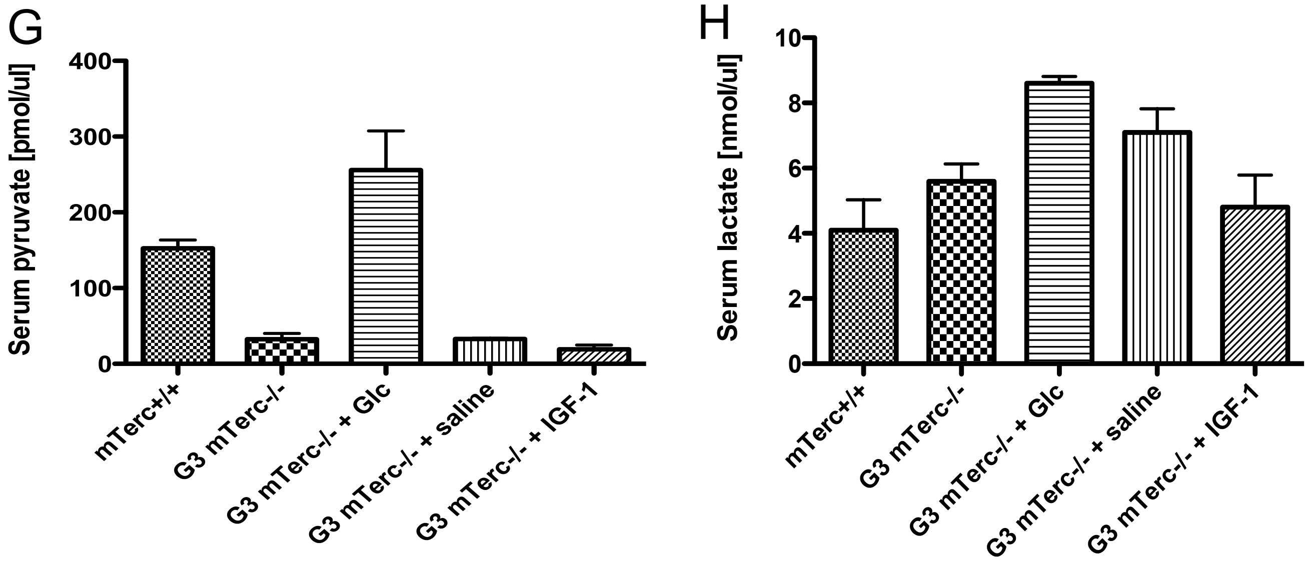

Supplementary Figure 4:
12-15 month old G3 *mTerc*$^{-/-}$ mice rescued with glucose-enriched diet were shifted to normal diet and separated into two groups: one saline-treated group (n=4 mice) and one IGF-1-treated group (n=5 mice).
A-C) Representative analysis of the immunohistochemistry staining in small intestine for **(A)** PCNA, **(B)** p53 and **(C)** p21 with and without IGF-1 supplementation.
D) Western Blot of phospho-p53, p21, γH2aX in pooled samples of the small intestine **(D)** of 12-15 month G3 *mTerc*$^{-/-}$ treated with saline and IGF-1. (n=4-5 mice per group).
E, F) Graphs show the relative citrate-synthase (CS)-**(E)** and Cytochrome-c-oxidase (COX)-expression **(F)** in liver homogenates of the respective groups (n=4-6 mice per group).
G, H) Assembled graphs of serum levels of **(G)** pyruvate and **(H)** lactate of all tested groups. (n=4-6 mice per group)

Supplementary Figure 5

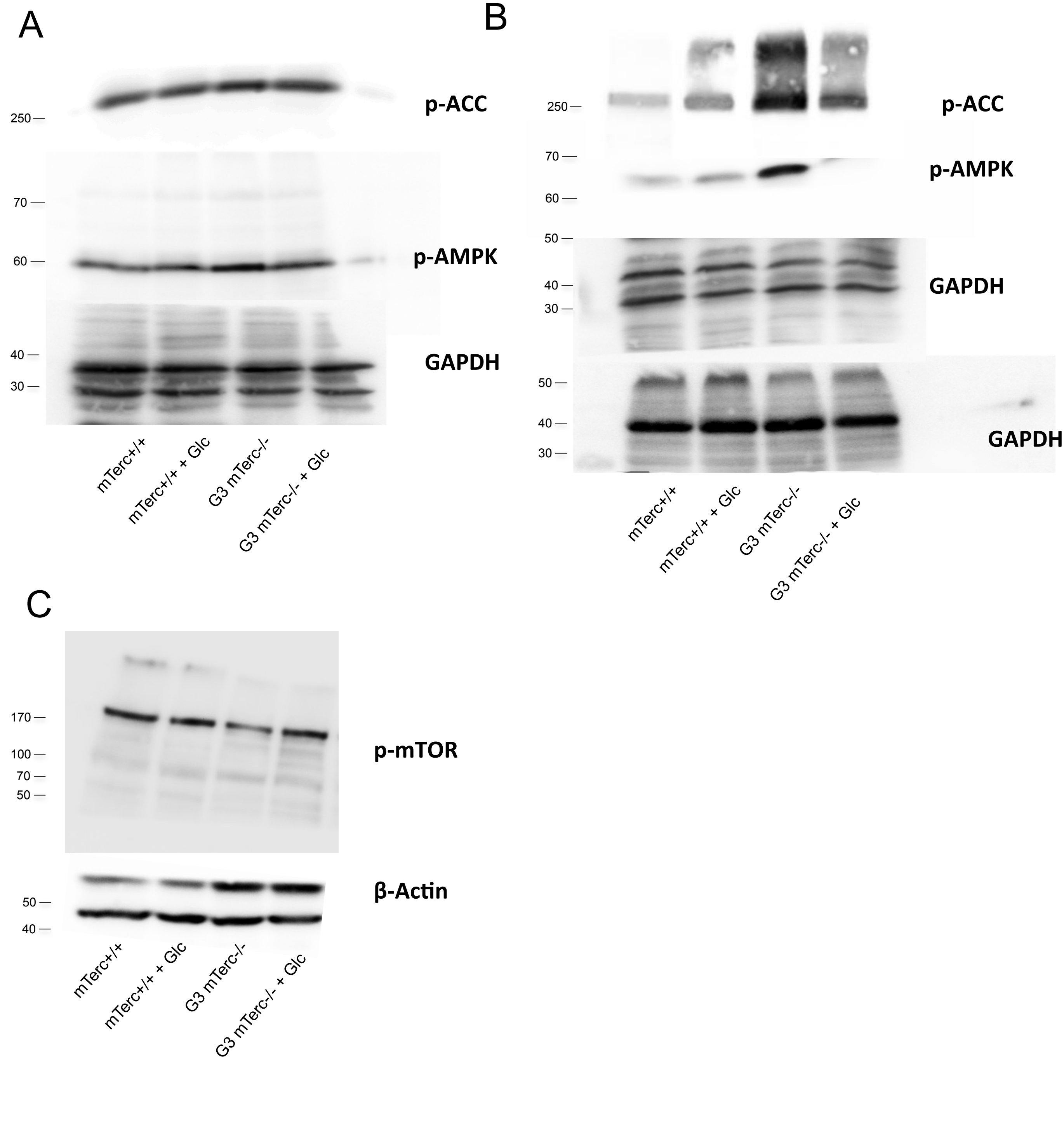

Supplementary Figure 5:
The figure shows uncropped western blots displayed in Fig. 3F **(C)**, as well as Fig. 6E **(A)** and Fig. 6F **(B)**
A,B) Western blot analysis of protein levels of phosphorylated-ACC (as a phorsphorylated-AMPK target) and phorsphorylated-AMPK in frozen liver **(A)** and skeletal muscle **(B)** extracts of the indicated groups (n=4-6 mice per group).
C) The Western Blot shows the different expressions of phosphorylated-mTOR in liver tissues of mice fed with ad libitum normal diet or high glucose diet (n=4-5 mice per group).

Supplementary Table 1

	Control diet	Glucose rich diet
**Energy content [MJ/kg]**	18.0	17.9
**Fat [%]**	30	27
**Protein [%]**	9	6
**Carbohydrates [%]**	61	67
- **starch [%]**	46.8	0
- **glucose [%]**	0	>50
- **oligosaccharides, dextrine [%]**	>10	>12

Supplementary Table 1:
The table shows the nutritional characteristics of the control and the high glucose diet fed to the different cohorts of mice.

Supplementary Table 2

Name	Forward	Reverse
PGC1a	5’ CCCTGCCATTGTTAAGACC 3’	5’ TGCTGCTGTTCCTCTTTTC 3’
PGC1ß	5’ GGACGCCAGTGACTTTGACT 3’	5’ TTCATCCAGTTCTGGGAAGG 3’
ERRa	5’ GCAGGGCAGTGGGAAGCTA 3’	5’ CCTCTTGAAGAAGGCTTTGCA 3’
NRF-1	5’ GAACTGCCAACCACAGTCAC 3’	5’ TTTGTTCCACCTCTCCATCA 3’
TFAM	5’ AATGTGGAGCGTGCTAAAAGC 3’	5’ GCTGAACGAGGTCTTTTTGGT 3’
p21	5’ TATTTAAGCCCCTCCCAACC 3’	5’ AGCTGGCCTTAGAGGTGACA 3’
Puma	5’ ATGGCGGACGACCTCAAC 3’	5’ AGTCCCATGAAGAGATTGTACATGAC 3’
COXI	5’ CTGAGCGGGAATAGTGGGTA 3’	5’ TGGGGCTCCGATT ATTAGTG 3’
ß-Globin	5’ GCACCTGACTGATGCTGAGAA 3’	5’ TTCATCGGCGTTCACCTTTCC 3’

Supplementary Table 2:

The table shows the different primer sets used for Real-Time PCR.
